# The household economic burden of eating disorders and adherence to treatment in Australia

**DOI:** 10.1186/s12888-014-0338-0

**Published:** 2014-11-29

**Authors:** Lauren Gatt, Stephen Jan, Naresh Mondraty, Sarah Horsfield, Susan Hart, Janice Russell, Tracey Lea Laba, Beverley Essue

**Affiliations:** The George Institute for Global Health, University of Sydney, PO Box M201, Missenden Road, Sydney, NSW 2050 Australia; Wesley Eating Disorder Centre, Wesley Hospital, Sydney, Australia; Eating Disorders Day Program, Royal Prince Alfred Hospital, Sydney, Australia

## Abstract

**Background:**

This study investigated the household economic burden of eating disorders and cost-related non-adherence to treatment in Australia.

**Methods:**

Multi-centre prospective observational study using a structured questionnaire. Ninety participants were recruited from two clinic settings in New South Wales, Australia and from the community using social media. The primary outcome measures were household economic burden of illness measured in terms of out-of-pocket expenditure, household economic hardship and cost-related non-adherence.

**Results:**

The pattern of out-of-pocket expenditure varied by diagnosis, with Bulimia Nervosa associated with the highest total mean expenditure (per three months). Economic hardship was reported in 96.7% of participants and 17.8% reported cost-related non-adherence. Those most likely to report cost-related non-adherence had a longer time since diagnosis. Cost-related non-adherence and higher out-of-pocket expenditure were associated with poorer quality of life, a more threatening perception of the impact of the illness and poor self-reported health.

**Conclusions:**

This study is the first to empirically and quantitatively examine the household economic burden of eating disorders from the patient perspective. Results indicate that households experience a substantial burden associated with the treatment and management of an eating disorder. This burden may contribute to maintaining the illness for those who experience cost-related non-adherence and by negatively influencing health outcomes. Current initiatives to implement sustainable and integrated models of care for eating disorders should strive to minimise the economic impact of treatment on families.

**Electronic supplementary material:**

The online version of this article (doi:10.1186/s12888-014-0338-0) contains supplementary material, which is available to authorized users.

## Background

There were estimated to be 914,000 people with eating disorders in Australia in 2012 [1]. The costs to society in terms of costs of care, productivity cost and deadweight loss (foregone productive opportunities to society associated with higher taxes due to illness) was $69 billion in 2012 [1]. The costs of treatment alone for eating disorders can pose a substantial economic burden. For example, in the Australian private hospital sector, the treatment of an episode of Anorexia is estimated to come second to the cost of cardiac artery bypass surgery [2]. For individuals, although universal health coverage and social safety nets exist, significant costs and potential economic hardship are still experienced by many with chronic and long-term illnesses [3-8]. While direct costs for most health care services are either fully or partially subsidised, the limited availability of specialist services for the treatment and management of eating disorders in the public hospital system forces many to seek private treatment. Consequently, substantial unbudgeted out-of-pocket costs and copayments can still be faced. In addition to these costs, other economic and social consequences are incurred by individuals and their households, through changes in education, employment, work participation and social engagement, including for family members who provide necessary yet unpaid informal care [2-9].

When people with chronic illness struggle to pay for healthcare, cost-related non-adherence with treatment, particularly prescription medications, occurs [10-12]. Lack of drug coverage has been found to strongly inhibit prescription drug use, even in Australia where most essential medications for chronic illnesses are heavily subsidized through a national health insurance system, Medicare [11-13]. Such as scheme covers, with a patient copayment, the cost of listed medicines (through the Pharmaceutical Benefits Scheme (PBS)) and medical services (through the Medical Benefit Scheme (MBS)). Exemption from co-payments is available when individuals hit a certain ‘safety net’ threshold, which is a defined level of expenditure per calendar year (in 2014, the PBS threshold is $1421 per year for general beneficiaries and $360 per year low income concession card holders; for MBS it is $1248 and $624 respectively per year).

In addition, poor mental health has also been consistently linked to cost-related non-adherence [10]. Given medications such as antidepressants and antipsychotics are often used to treat comorbid psychological conditions of eating disorders such as depression, and anxiety, it is likely the problem of cost-related non-adherence also extends to eating disorders. Cost-related non-adherence to medications and medical care for eating disorders has not been previously explored in the literature.

There is a shortage of published empirical studies quantifying the household economic burden of eating disorders globally, including in the Australian context. While there are published cost-of-illness studies that have estimated the societal costs, the emphasis has generally been on the health system burden and productivity losses and not the burden faced by individuals and their families [14,15]. In addition, studies tend to focus on one illness and so have not provided comparisons across broad diagnosis groups [16]. Given the stigma associated with such conditions and other challenges associated with recruitment, studies also tend to be small, qualitative or based on retrospective analyses of administrative data. Here we provide a more nuanced examination of the diverse economic impacts associated with treatment and management, with a particular focus on the impact on patients and their families. The aim of this study was to investigate the household economic burden of eating disorders in Australia and the relationship between such burden and health outcomes.

## Methods

We recruited English-speaking individuals who received treatment for any eating disorder in Sydney, Australia at Wesley Private Hospital and at the outpatient program at Royal Prince Alfred (RPA) Public Hospital. Given the challenges of recruiting research participants with an eating disorder, particularly given the stigma involved in such illness [17], we used a multifaceted recruitment strategy. An opt-in invitation and a study questionnaire were posted to all individuals who received treatment at Wesley Hospital from January 2009 to March 2011, and were sent by email with a link to an online version of the study documents to all individuals who received treatment at RPA Hospital from January 2010 to December 2012. Individuals either self-administered the questionnaire or completed it with a researcher. The questionnaire was re-sent, either by post or email to all non-respondents six weeks after the initial mailing and the remaining non-respondents at 10 weeks were re-contacted by telephone with a further reminder. In addition, current patients at each centre were invited to participate in the study and either self-administered the study questionnaire or completed it with the assistance of a researcher.

Recruitment information was also posted on two national consumer websites: The Centre for Eating and Dieting Disorders and the Butterfly Foundation and was posted on the Butterfly Foundation’s Facebook page. Interested individuals completed the study questionnaire over the phone with the assistance of a researcher.

The return of a completed questionnaire by post or online or completion over the telephone was understood to imply consent to participate in the study. Participants who completed the questionnaire in person with the assistance of a researcher provided written informed consent. This study was approved by the following Human Research Ethics Committees: Western Sydney Local Health District (2794); University of the Sydney (12623) and Sydney Local Health District (X12-0145).

The questionnaire was developed based on the authors’ previous work and included questions drawn from existing standardised tools. It covered the following domains: demographics; medical history; health outcomes; household economic circumstances and social resources (i.e. the number and type of social contacts) (Additional file 1: Table S1).

The primary outcome was the household economic burden of eating disorders measured using the following: out-of-pocket expenditure on medical and health-related expenses, household economic hardship (hardship hereafter) and cost-related non-adherence.

Participants reported out-of-pocket expenditure in the past three months for: prescription and non-prescription medications; medical and allied health care; hospitalisations; medical tests; medically-related transportation; home and self-care assistance; medical equipment and supplies; and special food requirements. The burden of out-of-pocket expenditure, measured as the proportion of household income spent on illness-related expenses in the past three months is also reported. Equivalised household income (income hereafter) was used for this outcome [18]. It makes adjustments to actual income to account for households of different size and composition.

Hardship was measured using a series of questions about financial stress (e.g. failure to pay living and medical expenses) and the use of dissaving actions in the previous 12 months. Dissaving behaviour is an action where spending is greater than income thereby reducing already accumulated savings, leading or borrowing to finance the expenditure [19]. Hardship was a dichotomous variable where a reported inability to pay or the use of a dissaving action was classed as hardship.

Cost-related non-adherence was measured using the following questions: *In the last 12 months have you: a) missed a medical appointment due to cost; b) not filled a prescription due to cost*. This was a dichotomous variable where a ‘Yes’ response to either question was defined as cost-related non-adherence.

### Health outcomes

- Quality of life, using the EuroQoL questionnaire, a measure of health-related quality of life [20]. A score between zero (worst health) and one (best health) was generated;
- Self-reported health, using a standard question from the Short Form 12 survey of health-related quality of life [21]: *How would you rate your health today?* Participants were provided with five discrete response options. We report the proportion of participants indicating ‘fair’ or ‘poor health’; and
- Illness perception, using the Brief Illness Perception Question (BIPQ), a nine item scale that assesses cognitive and emotional representations of illness [22]. A higher score indicates a more threatening perception of the impact of the illness.
- Eating Disorders Quality of Life Questionnaire [23]:
  The Eating Disorders Quality of Life (EDQOL) instrument, a disease-specific self-report questionnaire designed for patients with an eating disorder was also used to assess quality of life. A score between 0 and 4 is generated for each sub-scale (i.e. Financial; work and education). The lower the score, the better the quality of life rating.

We conducted descriptive analyses of frequencies for each component of hardship and calculated means, medians and distributions for total out-of-pocket expenditure and for each category of expenditure. Bivariate analyses, using the chi-square test and the independent t-test, were used to compare participants with and without cost-related non-adherence for categorical and continuous variables respectively. Logistic regression was used to identify the factors that were associated with cost-related non-adherence, beginning with a saturated model that included all explanatory variables that were associated with the outcome (P <0.25) in the univariate analysis (see Additional file 1: Table S2). Variables were assessed individually for significant contribution to the overall model (P < 0.05). Effect modification was checked between variables in the model to identify significant interactions (P < 0.01). The Hosmer and Lemeshow goodness-of-fit test was used to check the fit of the final model. In addition, the analysis of variance test was used to analyse the relationship between the economic outcomes and health outcomes. Data analysis was conducted using SPSS 21.

## Results

### Characteristics of the study population

**Figure 1:**
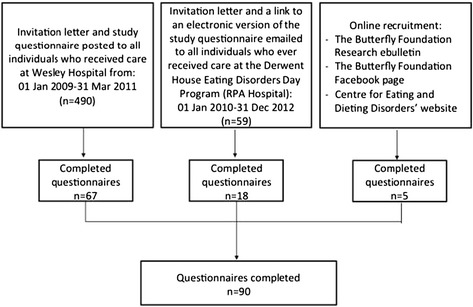
**Study flow chart.**

Figure 1 shows the flow of participants into the study. Ninety participants completed questionnaires. 54.4% of participants had a self-reported diagnosis of Anorexia Nervosa (AN), either purging or restricting subtype, 16.7% had a diagnosis of Bulimia Nervosa (BN) and 28.9% were diagnosed as either Binge Eating Disorder, Eating Disorder Not Otherwise Specified or did not know their diagnosis (Other hereafter) (Table 1). Although sex was not a basis for exclusion, 99% of respondents were female (Table 1). Participants recruited from Wesley Hospital were more likely to have the following characteristics: a diagnosis of AN, a hospital admission in the previous 12 months, qualified for the Medicare or Pharmaceutical Benefits Scheme safety net programs at the time of the interview, private health insurance and were less likely to be in paid employment. Participants were similar in all other characteristics.

**Table 1:** **Characteristics of the study population overall and by recruitment site** ^**a**^

**Personal and family information**	**Total**	**Private Hospital**	**Public Hospital**
	**(n = 90)**	**(n = 67)**	**(n = 18)**
Age, mean (±SD)	24.5 (SD = 8.3)	28.9 (SD:8.7)	27.3 (SD:6.9)
Female	89 /90 (98.9%)	66 (98.5%)	18 (100%)
English spoken at home	81/90 (90.0%)	60 (89.6%)	17 (94.4%)
*Marital status*			
Couple	21/89 (23.6%)	15 (22.4%)	6 (33.3%)
Single	68/89 (76.4%)	52 (77.6%)	12 (66.7%)
*Current living situation*			
Living with parents	42/90 (46.7%)	33 (49.3%)	7 (38.9%)
Living with spouse/partner	21/90 (23.3%)	16 (23.9%)	5 (27.8%)
Living alone	14/90 (15.6%)	10 (14.9%)	2 (11.1%)
Living in shared accommodation	12/90 (14.4%)	8 (11.9%)	4 (22.2%)
*Highest qualification completed or in the process of completing*			
Secondary school or lower	19/89 (21.3%)	16 (23.9%)	3 (16.7%)
University or TAFE	70/89 (78.7%)	51 (76.1%)	14 (77.8%)
*Current work status* ^*b*^			
Employed (full or part-time)	42/90 (46.7%)	30 (44.8%)	10 (55.6%)
Employed, on sick leave	7/90 (7.8%)	3 (4.5%)	3 (16.7%)
Unemployed	6/90 (6.7%)	4 (6.0%)	2 (11.1%)
Unemployed, due to medical reasons	22/90 (24.4%)	20 (29.9%)	0
Other	13/90 (14.4%)	10 (14.9%)	2 (11.1%)
*Medical information*			
Age when eating disorder was diagnosed, mean (±SD)	18.0 (SD = 4.9)	17.6 (SD:4.8)	19.9 (SD:5.1)
Years since diagnosis, mean (±SD)	10.6 (SD = 8.3)	11.2 (SD:8.6)	7.8 (SD:6.6)
BMI, mean (±SD)	19.4 (SD = 3.9)	19.1 (SD:4.2)	20.1 (SD:2.7)
*Eating disorder diagnosis, by broad category* ^*b,c*^			
Anorexia Nervosa	49/90 (54.4%)	45 (67.2%)	2 (11.1%)
Bulimia Nervosa	15/90 (16.7%)	11 (16.4%)	2 (11.1%)
Other	26/90 (28.9%)	11 (16.4%)	14 (77.8%)
*Psychological co-morbidity*			
Alcohol and/or substance abuse	16/88 (18.2%)	13 (19.7%)	2 (11.1%)
Anxiety disorders	50/88 (56.8%)	41 (62.1%)	7 (41.2%)
Bi-polar disorder	4/88 (4.5%)	3 (4.5%)	1 (5.9%)
Depression	77/88 (87.5%)	60 (90.9%)	14 (82.4%)
Psychosis	4/88 (4.5%)	3 (4.5%)	1 (5.9%)
Proportion with a hospital admission for the treatment of the eating disorder or associated complications in the past 12 months?^b^	50/89 (56.2%)	43 (64.2%)	4 (23.5%)
*Insurance status*			
Reached the Pharmaceutical Benefits Scheme (PBS) safety net threshold^b^	15/89 (16.9%)	15 (22.4%)	0
Reached the Medicare safety net threshold^b^	31/89 (34.8%)	28 (41.8%)	2 (11.8%)
Private health insurance^b^	78/89 (86.7%)	63 (94.0%)	12 (70.6%)
*Income*			
AUD$20,000 and under	9/89 (10.1%)	6 (9.0%)	1 (5.9%)
AUD$20,000-39,999	19/89 (21.3%)	16 (23.9%)	3 (17.6%)
AUD$40,000-59,999	7/89 (7.9%)	4 (6.0%)	3 (17.6%)
AUD$60,000-79,999	7/89 (7.9%)	4 (6.4%)	1 (5.9%)
AUD$80,000-99,999	7/89 (7.9%)	4 (6.4%)	3 (17.6%)
AUD$100,000 or more	22/89 (24.4%)	20 (29.9%)	1 (5.9%)
Don’t know/rather not answer	18/89 (20.2%)	13 (19.4%)	5 (29.4%)

Data are shown as frequency and proportion (%) or mean and standard deviation (±SD).

^a^Data are not shown separately for the subsample who were recruited online as n = 5.

^b^We found a significant difference in these variables by recruitment site, *P* < 0.05.

^c^Anorexia includes: Anorexia nervosa purging subtype and restricting subtype; Other includes: Binge Eating Disorder, Eating Disorder Not Otherwise Specified and Don’t know / not sure.

### Household economic outcomes

96.7% of participants reported experiencing hardship in the previous 12 months and this proportion was similar across diagnosis groups (Table 2). Paying for medical appointments was the most commonly reported source of financial stress (44.3%), followed by dental appointments (31.0%), utility bills (29.5%), medications (25.3%) and food (25.0%). Using savings that had been put aside for other purposes (61.4%) and seeking assistance from friends and family (45.3%) were the most commonly reported strategies used when faced with financial stress. 17.8% of participants reported cost-related non-adherence and this was more common among those with a diagnosis of AN (20.4%) or BN (20.0%) compared to those with a diagnosis otherwise classified (11.5%).

**Table 2:** **Summary of household economic outcomes in the study population**

	**Overall**		**Anorexia Nervosa**		**Bulimia Nervosa**		**Other**	
	**n = 90**		**n = 49**		**n = 15**		**n = 26**	
***Household economic outcomes***	**n**	**%**	**n**	**%**	**n**	**%**	**n**	**%**
**Economic hardship**	**87**	**96.7%**	**48**	**98.0%**	**14**	**93.3%**	**25**	**96.2%**
*Could not pay for*								
Utilities	26	29.5%	14	29.2%	4	26.7%	8	32.0%
Rent/mortgage	14	15.9%	8	16.7%	3	20.0%	3	12.0%
Car payments	19	21.6%	13	27.1%	1	6.7%	5	20.0%
Minimum credit card payment	19	21.6%	12	25.0%	2	13.3%	5	20.0%
Medications	22	25.3%	15	31.3%	3	20.0%	4	16.7%
Medical/health appointments	39	44.3%	22	45.8%	5	33.3%	12	48.0%
Health insurance	13	14.9%	7	14.9%	2	13.3%	4	16.0%
Dental appointments	27	31.0%	17	36.2%	2	13.3%	8	32.0%
Transport	14	16.3%	10	21.7%	1	6.7%	3	12.0%
Food	22	25.0%	13	27.1%	4	36.7%	5	20.0%
Did not attend medical appointments	30	34.5%	16	34.0%	5	33.3%	9	36.0%
Did not fill prescriptions	23	26.1%	15	31.3%	4	26.7%	4	16.0%
Unable to heat home	13	14.8%	8	16.7%	2	13.3%	3	12.0%
*Did you do use any of these strategies because you were short on money?*								
Reduced home loan payments	5	5.7%	1	2.1%	2	13.3%	2	8.0%
Used savings put aside for other purposes	54	61.4%	30	62.5%	8	53.3%	16	64.0%
Moved house	13	14.9%	8	17.0%	4	26.7%	1	4.0%
Increased credit card debt $1000 or more	26	29.9%	13	27.7%	4	26.7%	9	36.0%
Sought financial assistance from friends/family	39	45.3%	21	45.7%	8	53.3%	10	40.0%
Sought financial assistance from welfare or community organisation	20	23.0%	12	25.5%	3	20.0%	5	20.0%
Informal loan	14	16.1%	7	14.9%	3	20.0%	4	16.0%
Formal loan	9	10.3%	5	10.6%	1	6.7%	3	12.0%
Sold assets	13	14.9%	4	8.5%	4	26.7%	5	20.0%
Other	16	18.4%	10	21.3%	2	13.3%	4	16.0%
**Cost-related non-adherence**	**16**	**17.8%**	**10**	**20.4%**	**3**	**20.0%**	**3**	**11.5%**
**Spent greater than 10% of income on medical and health-related expenses (self-reported)**	**30**	**41.7%**	**17**	**44.7%**	**6**	**46.2%**	**7**	**33.3%**

**Figure 2:**
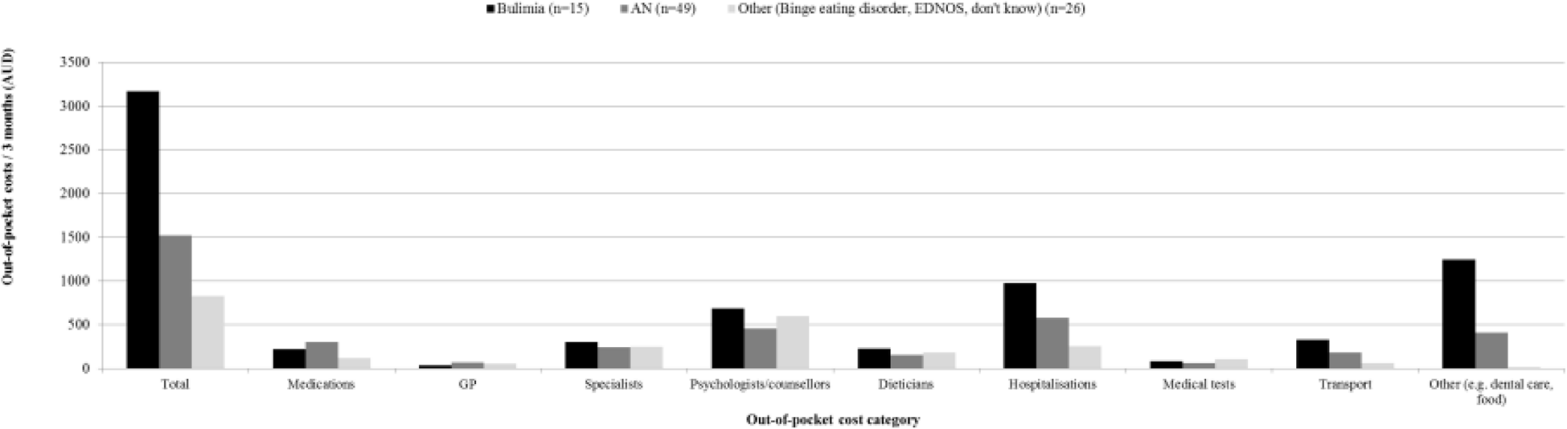
**Out-of-pocket cost for 3 months by disease and expenditure category.**

Participants with a BN diagnosis reported spending double the amount out-of-pocket in the previous three months compared to those with an AN diagnosis (BN: AUD$3175 versus AN: AUD$1525) and this was consistent for most expenditure categories (Figure 2). The main expenditure categories for all participants were hospitalisations (AN: AUD$580; BN: AUD$977; Other: AUD$255), psychologist and counsellor appointments (AN: AUD$456; BN: AUD$691; Other: AUD$600), specialist appointments (AN: AUD$243; BN: AUD$304; Other: AUD$249) and medications (AN: AUD$300; BN: AUD$222; Other: AUD$123). At these levels of expenditure, households spent a mean of 20.7% (SD:33.2; median: 7.5, IQR:17.1) of income on medical and health-related expenses over a three month period. The burden of out-of-pocket expenditure was highest among those with BN (AN:22.9% (SD:33.8); BN:35.6% (SD:48.1); Other: 7.4% (SD:8.3); F = 3.3, *P* = 0.04).

### Determinants of cost-related non-adherence

Table 3 summarises the determinants of cost-related non-adherence in the past 12 months (see Additional file 1: Table S2 for the univariate results). The time since diagnosis was the only significant determinant, indicating that for every additional year since diagnosis with an eating disorder, participants were 1.15 times more likely to report cost-related non-adherence (95% CI: 1.02-1.30, *P* = 0.02).

**Table 3:** **Determinants of cost-related non-adherence**

	**Unadjusted point estimate**		**Adjusted point estimate** ^**a**^	
**Characteristic**	**OR (95% CI)**	**P-Value**	**OR (95% CI)**	**P-Value**
Time since diagnosis (years)	1.06 (1.00–1.12)	0.05	1.15 (1.02–1.30)	0.02
Number of hospital admissions in previous 12 months	1.59 (1.08–2.35)	0.02	1.44 (0.98–2.11)	0.06
Quality of life (EQ-5D)	0.08 (0.005–1.13)	0.06	0.30 (0–6.24)	0.20

^a^The adjusted model was built using backward selection of all variables associated with the outcome variable at the level of P <0.25 in univariate analysis (See Additional file 1: Table S2). Hosmer and Lemeshow goodness-of-fit test: χ^2^: 6.12, p = 0.63.

### Relationship between economic and health outcomes

For both economic outcomes, there was a negative relationship with quality of life, participants’ perceptions of the impact of the illness and self-reported health. Each health outcome worsened with higher out-of-pocket expenditure and poorer health outcomes were reported by those with cost-related non-adherence (Table 4).

**Table 4:** **Determinants of catastrophic health expenditure**

	**Unadjusted point estimate**		**Adjusted point estimate** ^**a**^	
**Characteristics**	**OR (95% CI)**	**P-Value**	**OR, 95% CI**	**P-Value**
Time since diagnosis (years)	1.05 (0.99-1.11)	0.092	1.15 (1.01-1.31)	0.04
Number of hospital admissions in previous 12 months	1.39 (0.91-2.12)	0.133	1.54 (1.01-2.36)	0.05
Number of hardship indicators	1.11 (0.99-1.24)	0.062	0.76 (0.56-1.03)	0.08
Reached Medicare Safety Net		0.034		0.098
Yes	1.0		1.0	
No	0.12 (0.021-0.62)		0.059 (0.004-0.90)	
Don’t know	0.46 (0.16-1.32)		1.23 (0.23-6.46)	

^a^The adjusted model was built using backward selection of all variables associated with the outcome variable at the level of P <0.25 in univariate analysis (See Additional file 1: Table S2). Hosmer and Lemeshow goodness-of-fit test: χ^2^:6.40, p = 0.60.

## Discussion

To the authors’ knowledge, this is the only study to comprehensively and quantitatively examine the household economic burden associated with eating disorders across multiple sites. We found high rates of hardship, high out-of-pocket expenditure and a high out-of-pocket cost burden in the study population. 97% of participants reported at least one incident of hardship in the previous 12 months. Over 40% of participants spent greater than 10% of their household income on illness-related costs, a level of expenditure that has been defined as ‘catastrophic’ in other studies [24,25]. In comparison, in other chronic disease populations in Australia, only 12% and 5% of the study population spent greater than 10% and 20% of their income respectively on illness-related costs [26]. The impacts of this burden were reinforced by the other outcome measures, with cost-related non-adherence reported by 18% of participants and the finding of poorer health outcomes among those experiencing a great economic burden.

Unlike other chronic conditions, the model of care for managing eating disorders in Australia largely sits outside of the primary health care setting and includes a mix of specialist eating disorder programs, most of which are offered in private settings. Other than through admission to hospital for acute treatment for severe episodes, the availability of treatment through the public system is limited. Private health insurance policies generally pay for inpatient care but coverage is variable for services provided in an outpatient or day program setting. A consequence of this, as found in this study, is that patients who access outpatient care as opposed to those admitted as inpatients tend to experience a greater burden.

The Australian National Framework for Eating Disorders has called for the development of a sustainable and integrated service model and as part of this, a review of Medicare items and private health insurance funding to ensure that the current financing models better support the diverse continuums of care required by patients with different eating disorders [9]. In light of the findings of this study, this would also help to ensure access to optimal and potentially cost-effective care and help ensure that the current models of care do not continue to undermine the equity objectives of the Australian health care system by creating disparities in access, utilisation and economic burden.

The results of this study supported the hypothesis that the amount and composition of out-of-pocket expenditure would vary by diagnosis. This is likely a function of the differences between the disorders, which may require different models of treatment. For example, the majority of the sample had private health insurance with hospital coverage. Patients with AN are more likely than patients with other eating disorders to require lengthy inpatient admissions to recover weight. As a public patient in a public hospital this would be free of charge, as a private patient a significant portion of treatment costs would nonetheless be covered by private health insurance. However, those with BN generally undergo outpatient treatment or receive care in day programs, for which the coverage of services by private health insurers can be variable. This coupled with transport costs to receive this treatment may provide some explanation as to why patients with BN had significantly higher out-of-pocket expenditures.

Cost-related non-adherence found in this study population suggests that the household economic burden associated with eating disorders may contribute to maintaining the illness by preventing participation in ongoing treatment. This identifies a need to improve the affordability of services available to patients. For example, within Australia’s public system, Medicare’s Better Access Initiative provides rebates for up to 10 visits per calendar year with an allied mental health service (e.g. psychologist), which falls short of the recommended guidelines of 40 visits for patients with AN and 20 visits for those with BN [27]. In this study, participants reported spending an average of AUD$535 out-of-pocket (in the past 3 months) on psychologist appointments. Given the high prevalence of psychological comorbidities [16] and moderate rates of full recovery experienced by patients with an eating disorder [28-30], the development of strategies to minimise cost-related non-adherence may help to break the cycle in which the economic burden of illness on households prevents optimal long term treatment and in turn, negatively influences health outcomes.

These findings should be interpreted in the context of the study’s limitations. First, this study was conducted with a mostly urban dwelling population who had received treatment on at least one previous occasion. In the absence of other empirical and quantitative data, this study provides important insights into the cost-pressures associated with treatment of an eating disorder. However, it is likely that individuals residing in rural and remote settings face additional economic barriers associated with accessing care as most specialist services are provided in urban settings in Australia. We have also missed individuals who have forgone treatment, either due to cost or other reasons (e.g. stigma). Because the sample is not likely to be representative, it was not possible to derive generalisable estimates of the prevalence of economic hardship outcomes in this study. The focus was on the factors that were associated with this set of outcomes. Second, 29% of the sample indicated a diagnosis ‘not otherwise specified’. Given the likely heterogeneity in this subgroup, it is also not possible to generalise from the findings of this subgroup. Third, despite using a multidimensional recruitment strategy, this study had a low participation rate. This is not uncommon in studies in eating disorder populations [17] however, we cannot rule out the possibility that this study may be under-represented by those with poorer outcomes who may be less likely to participate in research. Fourth, the outcomes were measured at one point in time so it is not possible to ascertain with certainty the direction of relationships reported. Longitudinal research in a larger representative sample of patients will help to elucidate the determinants and potential ameliorating factors of this burden.

This study has shown that hardship may be a disease maintaining factor as people may fail to seek the necessary care for economic reasons and this can impact on health outcomes. A better understanding of the factors underlying hardship has the potential to break this cycle.

## Conclusions

This research raises important policy implications. The current models of funding for the treatment of eating disorders do not encourage management of patients in the community or in outpatient settings because the public system offers limited treatment options beyond admission for severe, acute episodes. Private health insurance tends to provide only limited reimbursement outside private hospital admission. This pattern of funding discourages upstream management of illness, does little to prevent exacerbation of illnesses and leads to a model of care based on high cost acute management. This inevitably creates inequities in access to treatment based on socioeconomic status, access to health insurance and type of condition. This research underscores the need for more sustainable and affordable treatment models within the public and private systems to not only improve health outcomes, but to mitigate the substantial household economic burden experienced by patients undergoing care.

## Additional file

Additional file 1:
**Supplementary methods (** **Table S1:** **Summary of domains included in the study questionnaire;** **Table S2:** **Univariate results for characteristics associated with cost-related non-adherence).**
