# Supplementary material for: The household economic burden of eating disorders and adherence to treatment in Australia
Source: BMC Psychiatry. 2014 Nov 29;14:338. doi: 10.1186/s12888-014-0338-0 (PMC4262969; doi:10.1186/s12888-014-0338-0)
Supplement: Additional file 1: — Supplementary methods ( Table S1: Summary of domains included in the study questionnaire; Table S2: Univariate results for characteristics associated with cost-related non-adherence). [file 12888_2014_338_MOESM1_ESM.docx]

**Supplementary Methods:**

Table S1: Summary of domains included in the study questionnaire^a^

| Questionnaire domain |
| --- |
| *Personal and family information*(1) |
| Date of birth |
| Sex |
| Ethnicity |
| Language spoken at home |
| Marital status |
| Current living arrangements |
| Household composition |
| *Medical information* |
| Age at diagnosis |
| Current weight and height |
| Diagnosis |
| Psychological comorbidity |
| Health service utilisation |
| Self-rated health (SF-12) |
| Quality of life (4,5) |
| Illness perceptions (6) |
| *Education and employment* |
| Education status |
| Employment status |
| Main lifetime occupation |
| *Social contacts* |
| Number and type of social contacts |
| Perceptions of risk and trust (2) |
| *Household financial information^b^*(2, 3) |
| Private health insurance status |
| Access to financial resources |
| Financial stress |
| Dissaving actions |
| Out-of-pocket expenditure |
| Safety net qualification |
| Government assistance |
| Income |
| Perception of economic well-being |
|  |

^a^Where a caregiver was nominated (n=35), Part B of the questionnaire was completed by caregivers. The caregiver questionnaire included the same domains and an additional domain that assessed caregiver impact.

^b^ This section was completed by participants who indicated that they were financially independent. Where participants indicated that they had a caregiver and were not financially independent, this section was completed by the caregiver.

Table S2: Univariate results for characteristics associated with cost-related non-adherence

|  |  | Cost-related non-adherence | |  |
| --- | --- | --- | --- | --- |
| Characteristics | Total  n=90 | Yes  (n=16) | No  (n=74) | *Univariate P-value* |
| Age (years, mean ±SD) | 24.5 (SD:8.3) | 30.8 (SD:10.3) | 27.8 (SD:7.8) | 0.22 |
| Female | 89 /90 (98.9%) | 16/16 (100.0%) | 73/74 (98.6%) | 0.64 |
| Current living situation |  |  |  | 0.024 |
| Living with parents | 42/90 (46.7%) | 4/16 (25.0%) | 38/74 (51.4%) |  |
| Living with spouse | 21/90 (23.3%) | 5/16 (31.3%) | 16/74 (21.6%) |  |
| Living alone | 14/90 (15.6%) | 6/16 (37.5%) | 8/74 (10.8%) |  |
| Living in shared accommodation | 12/90 (14.4%) | 1/16 (6.3%) | 12/74 (16.2%) |  |
| Diagnosis |  |  |  | 0.61 |
| Anorexia Nervosa | 49/90 (54.4%) | 10/16 (62.5%) | 39/74 (52.7%) |  |
| Bulimia Nervosa | 15/90 (16.7%) | 3/16 (18.8%) | 12/74 (16.2%) |  |
| Other^a^ | 26/90 (28.9%) | 3/16 (18.8%) | 23/74 (31.1%) |  |
| Time since diagnosis (years, mean ±SD) | 10.6 (SD:8.3) | 14.4 (SD:10.7) | 9.7 (SD:7.4) | 0.038 |
| Number of psychological comorbidities (mean, ±SD) | 1.8 (SD:1.0) | 1.9 (SD:0.7) | 1.7 (SD:1.0) | 0.61 |
| Number of hospital admissions in previous 12 months^b^ (mean, ±SD) | 2.4 (SD:2.1) | 4.3 (SD:2.9) | 1.9 (SD:1.6) | 0.036 |
| Quality of life |  |  |  |  |
| EuroQoL^c^ | 0.57 (SD:0.21) | 0.48 (SD:0.20) | 0.60 (SD:0.21) | 0.057 |
| EDQoL^d^ |  |  |  |  |
| Financial scale impact, mean (±SD) | 1.47 (SD:1.05) | 2.31 (SD:0.86) | 1.28 (SD:0.99) | <0.0001 |
| Work/education impact, mean (±SD) | 1.67 (SD:0.83) | 2.08 (SD:0.63) | 1.58 (SD:0.85) | 0.029 |
| Illness perception, mean (±SD)^e^ | 45.9 (SD:13.2) | 52.0 (SD: 7.9) | 44.6 (SD:13.8) | 0.008 |
| Out-of-pocket cost burden (median, 95% CI) | 7.5 (5.1-12.4) | 39.6 (SD:44.2) | 16.2 (SD:28.6) | 0.08 |
| Caregiver | 35/90 (38.9%) | 3/16 (18.8%) | 32/74 (43.2%) | 0.068 |
| Household income affected by eating disorder | 69/90 (76.7%) | 16/16 (100%) | 53/73 (72.6%) | 0.017 |
| Private health insurance | 78/89 (86.7%) | 12/16 (75.0%) | 66/73 (90.4%) | 0.090 |
|  |  |  |  |  |

^a^Other included the following broad diagnosis categories: ‘Binge eating’, ‘Eating Disorder not elsewhere classified’ and ‘don’t know diagnosis’

^b^ Hospital admissions was defined as at least one night admission to hospital

^c^ Measured using the EuroQoL health-related quality of life tool (4)

^d^ Measured using the Eating Disorder Quality of Life tool (5)

^e^ Measured using the Brief Illness Perception Questionnaires (6)

**References:**

1. Banks E, Redman S, Jorm L, et al. Cohort profile: the 45 and up study. Int J Epidemiol. 2008;37:941-947.
2. Australian Bureau of Statistics. 2010 General Social Survey: Summary results. 2011; Canberra: ABS. Cat no. 4159.0.

1. Essue B, Kelly P, Roberts M, et al. We can't afford my chronic illness! The out-of-pocket burden associated with managing chronic obstructive pulmonary disease in western Sydney, Australia. J Health Serv Res Policy. 2011;16(4):226-231.

1. Brooks R. EuroQol: the current state of play. Health Policy. 1996;37(1):53-72.
2. Engel SG, Wittrock DA, Crosby RD, et al. Development and psychometric validation of an eating disorder-specific health-related quality of life instrument. Int J Eat Disord. 2006;39(1):62-71.

1. Broadbent E, Petrie KJ, Main J, et al. The brief illness perception questionnaire. J Psychosom Res. 2006;60(6):631-637.
